# Comparative population genetics of mimetic *Heliconius *butterflies in an endangered habitat; Brazil's Atlantic Forest

**DOI:** 10.1186/1471-2156-12-9

**Published:** 2011-01-20

**Authors:** Priscila Albuquerque de Moura, Swee-Peck Quek, Márcio Z Cardoso, Marcus R Kronforst

**Affiliations:** 1 Programa de Pós-graduação em Ecologia, Universidade Federal do Rio Grande do Norte, Natal, RN, Brazil; 2 FAS Center for Systems Biology, Harvard University, Cambridge MA 02138, USA; 3 Departamento de Botânica, Ecologia e Zoologia, Universidade Federal do Rio Grande do Norte, Natal, RN, Brazil

## Abstract

**Background:**

Brazil's Atlantic Forest is a biodiversity hotspot endangered by severe habitat degradation and fragmentation. Habitat fragmentation is expected to reduce dispersal among habitat patches resulting in increased genetic differentiation among populations. Here we examined genetic diversity and differentiation among populations of two *Heliconius *butterfly species in the northern portion of Brazil's Atlantic Forest to estimate the potential impact of habitat fragmentation on population connectivity in butterflies with home-range behavior.

**Results:**

We generated microsatellite, AFLP and mtDNA sequence data for 136 *Heliconius erato *specimens from eight collecting locations and 146 *H. melpomene *specimens from seven locations. Population genetic analyses of the data revealed high levels of genetic diversity in *H. erato *relative to *H. melpomene*, widespread genetic differentiation among populations of both species, and no evidence for isolation-by-distance.

**Conclusions:**

These results are consistent with the hypothesis that the extensive habitat fragmentation along Brazil's Atlantic Forest has reduced dispersal of *Heliconius *butterflies among neighboring habitat patches. The results also lend support to the observation that fine-scale population genetic structure may be common in *Heliconius*. If such population structure also exists independent of human activity, and has been common over the evolutionary history of *Heliconius *butterflies, it may have contributed to the evolution of wing pattern diversity in the genus.

## Background

Landscape structure has a fundamental influence on the distribution of populations, affecting their demography and genetics [1]. Some populations may be found continuously distributed while others are patchily distributed across their range, both of which may ultimately lead to some degree of genetic differentiation. Such geographic patterns of genetic variation reflect both historical processes, such as natural selection, and contemporary gene flow [2].

Gene flow determines the potential for genetic differentiation among populations and for local adaptation and the spread of novel adaptations [3,4]. In butterflies, as in other organisms, the nature and extent of gene flow is largely dependent on the mobility of individuals. Species with high vagility may disperse over large distances and therefore have extensive gene flow over large areas resulting in more homogeneous populations [5-8], whereas in species with low vagility, the effect of restricted dispersal will be evident at fine spatial scales [9-11]. Furthermore, gene flow may also be affected by a variety of ecological factors such as mating habits, gender-biased dispersal, diet specialization, habitat and population persistence, environmental factors and geographic distance [12].

Extensive studies using molecular markers on butterflies have shown how fragmentation leads to the reduction of gene flow among populations in different habitat patches and increases genetic differentiation among populations [9,13-15]. In fact, intra- and inter-population genetic variability is generally more affected in small patches of habitat and in small populations [6,16]. Fragmentation may even affect species with high vagility [8,11], leading to a decrease in genetic diversity due to reduced gene flow between populations.

**Figure 1:**
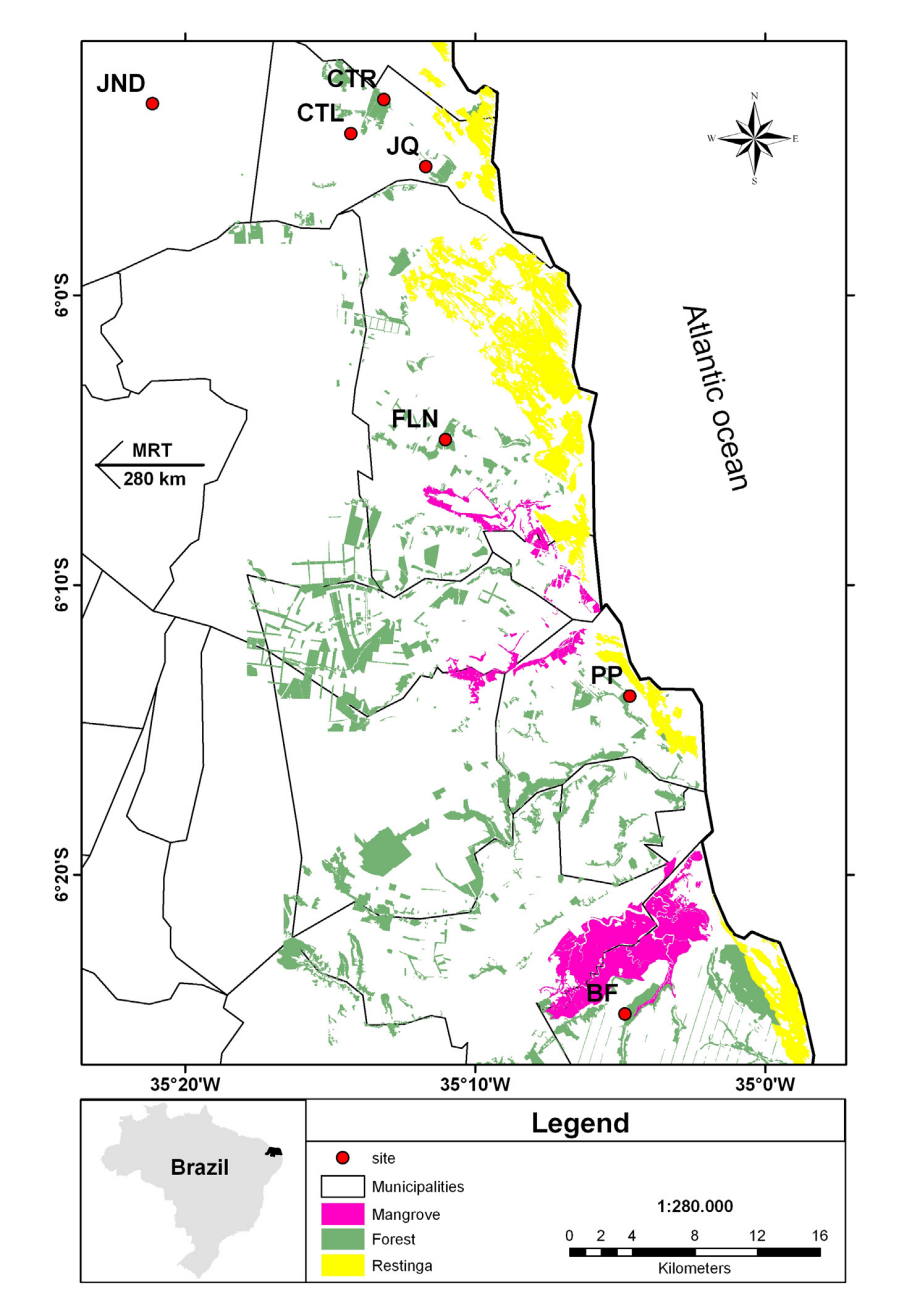
**Location of *Heliconius *collecting sites in Rio Grande do Norte, Brazil, relative to patches of undisturbed habitat**.

However, even though information on the relationship between fragmentation and genetic diversity in butterflies is available, many of these studies have been conducted with temperate species [6,14,17,18]. To date, few such studies have been conducted with tropical species. One tropical region of particular interest for this type of study is Brazil's Atlantic Forest (Figure 1), a highly fragmented biodiversity hotspot that has a high level of species endemism [19], including butterflies [20,21]. After more than 500 years of intensive human occupation, less than 7% of the original forest remains [22] and this region is now considered a priority area for conservation [23].

*Heliconius *butterflies are a well-studied group of tropical organisms [24-30], commonly found in New World tropical and subtropical forests [31]. Mark-recapture studies of *Heliconius *have shown that populations remain fairly stable over time, usually at low densities [26]. After a brief period of dispersal, *Heliconius *adults establish themselves in areas where they remain for the rest of their lives [32]. Thus, populations of *Heliconius *are organized in moderately sedentary units, with little movement of individuals, apparently as a result of home range behavior [32]. These behaviors suggest that gene flow among geographically separate populations may be low.

Yet, little is known about geographical structure of *Heliconius *populations and the effects of fragmentation on population connectivity. Most studies have employed mark-recapture methods to infer population structure [32-39], but some evidence from molecular markers is available. For instance, Kronforst & Fleming [7] analyzed population genetic structure of the highly vagile species *Heliconius charithonia *using allozymes and found low genetic diversity and no evidence of genetic subdivision in south Florida. Additional studies with allozymes have found little evidence for genetic differentiation in other *Heliconius *species [40-42]. In contrast, Kronforst & Gilbert [43] found evidence for extensive genetic differentiation and isolation by distance across multiple *Heliconius *species in Costa Rica using AFLP markers.

Population genetic structure in *Heliconius *also has important implications for the evolutionary dynamics of mimicry. *Heliconius erato *and *H. melpomene *are distantly-related species belonging to different clades within the genus [24,44]. These two species are Müllerian mimics of one another throughout their shared range of Central and South America but they have both diversified, in parallel, into over 20 geographic wing pattern races. Historically, the co-mimetic radiations of *H. erato *and *H. melpomene *were thought to have occurred in parallel across time and space, possibly facilitated by Pleistocene rainforest refugia [31]. However, data from a variety of sources, including modern population genetic data [45-47], support an alternative 'mimetic advergence' hypothesis [48], with *H. erato *radiating first and establishing the diversity of wing patterns which *H. melpomene *later evolved to mimic. A remaining issue then is how *H. erato *originally diversified, given the strong stabilizing selection expected on warning coloration. One hypothesis is that stochastic events in local populations have occasionally allowed novel phenotypes to drift over the frequency threshold necessary to become a learned, and hence protected, warning pattern [49,50] and this may be playing out as part of a larger "shifting-balance" type process [51-53]. This process requires small local population sizes as well as drift, and existing molecular data provide mixed evidence as to whether these conditions are generally met in *Heliconius*; allozymes have generally shown little structure while AFLPs have revealed more.

Our current analysis of population genetic structure in *Heliconius *was hence motivated by two factors. First, we were interested in determining the potential impact of the highly fragmented habitat along Brazil's Atlantic Forest on population connectivity in butterfly species with home range behavior. Second, we were interested in whether replicate analyses of population genetic structure in different geographic regions, and with a diversity of molecular markers, provide evidence for or against substantial population genetic structure in *Heliconius*. The generality of this phenomenon has important implications for the potential role of drift in color pattern diversification, as long as it has also occurred throughout the evolutionary history of *Heliconius *butterflies and independent of human activity. To address these questions, we analyzed the population genetic structure of co-mimics *H. erato *and *H. melpomene *throughout a portion of Brazil's Atlantic Forest using three types of molecular markers; microsatellites, AFLPs, and mitochondrial DNA sequences. Our results reveal substantial genetic differentiation among populations and intriguing differences between species and among molecular markers, reflecting the unique population biology of our study system and its geographic context.

## Results and Discussion

Brazil's Atlantic Forest is a biodiversity hotspot endangered by extreme deforestation. To examine the potential impact of this habitat fragmentation on butterfly dispersal, we measured genetic diversity and differentiation among remnant forest patches for two co-mimetic *Heliconius *butterfly species, *H. erato *and *H. melpomene*. Our analysis included multiple populations of both species and three distinct classes of molecular markers, allowing us to compare and contrast patterns across species, populations, and types of molecular data.

### Genetic Diversity

Across all marker types, *H. erato *displayed greater genetic diversity than *H. melpomene *(Table 1), which is consistent with previous genetic comparisons between the two species [43,45,47,54]. Elevated genetic diversity in *H. erato*, relative to *H. melpomene*, is commonly attributed to the fact that *H. erato *is generally more abundant than *H. melpomene *[26,48,55,56], which could result in a larger effective population size. Another potential contributing factor is that the geographic radiation of *H. erato *predates that of *H. melpomene *[45], allowing more time for the accumulation of standing genetic variation. It is unclear whether these explanations may apply to our sampling locations however. For instance, our collections revealed that *H. melpomene *is as abundant, or more abundant, than *H. erato *across most of our collecting sites. In addition, recent data suggest that *H. melpomene *may have originated in coastal Brazil while *H. erato *colonized this region after originating in western South America [47], which may mean that the two species have occupied this area for similar lengths of time. Regardless of the cause, our data show that *H. erato *is 14 times more variable than *H. melpomene *at mtDNA, seven times more variable at AFLP markers, and 1.3 times more variable at microsatellite loci (Table 1).

**Table 1:** Summary population genetic statistics across species and molecular markers.

	Markers (N)		Diversity		***F***_***ST***_		IBD (Mantel *r*)	
Dataset	*H. erato*	*H. melpomene*	*H. erato*	*H. melpomene*	*H. erato*	*H. melpomene*	*H. erato*	*H. melpomene*
**msats**	7 loci	5 loci	*H*_*O *_= 0.594	*H*_*O *_= 0.456	0.011	0.020	-0.168	-0.011
	(136 alleles)	(31 alleles)	(0.141 sd)	(0.107 sd)	(*P *= 0.160)	(*P *= 0.002)	(*P *= 0.280)	(*P *= 0.520)
**AFLP**	1144 loci	1144 loci	0.368	0.053	0.020	0.086	-0.193	0.205
	(1121 polys)	(487 polys)	(0.113 sd)	(0.106 sd)	(*P *< 10^-4^)	(*P *< 10^-4^)	(*P *= 0.116)	(*P *= 0.189)
**mtDNA**	1600 bp	1600 bp	π = 0.0086	π = 0.0006	0.145	0.017	0.094	-0.202
	(81 SNPs)	(15 SNPs)	(0.0043 sd)	(0.0005 sd)	(*P *< 10^-4^)	(*P *= 0.094)	(*P *= 0.335)	(*P *= 0.274)

### Differentiation and isolation-by-distance

Our analyses revealed widespread genetic differentiation in both species but substantial variation across marker types. Overall, *H. erato *displayed significant genetic differentiation at both AFLPs and mtDNA but not microsatellites while *H. melpomene *displayed significant genetic differentiation at microsatellites and AFLPs but not mtDNA (Table 1). Pairwise comparisons among populations revealed similar patterns. For instance, based on microsatellites, seven population comparisons were significant in *H. melpomene *while only two were significant in *H. erato *(Table 2). With AFLPs, virtually all pairwise comparisons were significant in both species (Table 3). Finally, mtDNA revealed 13 significant comparisons in *H. erato *and only four in *H. melpomene *(Table 4). Interestingly, mtDNA revealed much more genetic differentiation in *H. erato *than did the nuclear markers, suggesting females may be more sedentary than males in this species. While genetic differentiation appears to be widespread in both species, none of the tests for isolation-by-distance were significant (Table 1). This indicates that geographic distance alone is not a good indicator of genetic distance among populations.

**Table 2:** Pairwise population *F*_*ST *_values based on microsatellite data; *H. erato *comparisons below diagonal, *H. melpomene *above diagonal.

	MRT	JND	CTR	CTL	JQ	FLN	PP	BF
MRT	0	-	-	-	-	-	-	-
JND	0.0226	0	0.0077	0.0440*	0.0190	0.0093	0.0239	0.0278
CTR	0.0083	0.0244**	0	0.0312*	0.0119	-0.0015	0.0317*	-0.0060
CTL	0.0051	0.0061	0.0154	0	0.0139	0.0248*	0.0334*	0.0183
JQ	0.0029	0.0173*	0.0154	0.0094	0	0.0245*	0.0291*	0.0260
FLN	-0.0039	0.0046	0.0044	0.0007	0.0016	0	0.0195	0.0090
PP	0.0046	0.0163	0.0152	0.0104	0.0160	-0.0095	0	0.0218
BF	0.0011	-0.0007	0.0108	0.0060	0.0056	-0.0097	-0.0023	0

*P*-values are indicated as follows: * P < 0.05, ** P < 0.01, *** < 0.001, (no asterisk) = not significant.

**Table 3:** Pairwise population *F*_*ST *_values based on AFLP data; *H. erato *comparisons below diagonal, *H. melpomene *above diagonal.

	MRT	JND	CTR	CTL	JQ	FLN	PP	BF
MRT	0	-	-	-	-	-	-	-
JND	0.01832**	0	0.13739***	0.06361***	0.03481**	0.08283***	0.04566***	0.12646***
CTR	0.01117*	0.02969***	0	0.04576***	0.12178***	0.11645***	0.10596***	0.06254***
CTL	0.01519*	0.01937***	0.02173***	0	0.07282***	0.08292***	0.04716***	0.07188***
JQ	0.01704***	0.01656***	0.02653***	0.02037***	0	0.07318***	0.074***	0.18178***
FLN	0.04009***	0.02507*	0.03515***	0.02235*	0.02294**	0	0.03954***	0.16888***
PP	0.02068***	0.01496***	0.03057***	0.01615***	0.00698**	0.01170	0	0.12302***
BF	0.00223*	0.01850*	0.01383*	0.01485*	0.01445**	0.05357***	0.02061**	0

*P*-values are indicated as follows: * P < 0.05, ** P < 0.01, *** < 0.001, (no asterisk) = not significant.

**Table 4:** Pairwise population *F*_*ST *_values based on mtDNA sequence data; *H. erato *comparisons below diagonal, *H. melpomene *above diagonal.

	MRT	JND	CTR	CTL	JQ	FLN	PP	BF
MRT	0	-	-	-	-	-	-	-
JND	0.3290**	0	-0.0428	-0.0582	0.0665	-0.0229	-0.0456	-0.0706
CTR	0.1519*	0.0129	0	-0.0252	0.0800**	0.0074	-0.0082	-0.0305
CTL	0.4578***	-0.0160	0.0512	0	0.0852**	0.0006	-0.0181	-0.0425
JQ	0.1709*	0.0059	-0.0267	0.0564	0	0.0167	0.1105**	0.0784*
FLN	0.0441	0.4681**	0.2522*	0.5880**	0.3043*	0	0.0283	-0.0179
PP	-0.0355	0.2651**	0.1282*	0.3519***	0.1322*	0.0615	0	-0.0284
BF	0.2030*	-0.0308	-0.0223	0.0381	-0.0008	0.3039	0.1520	0

*P*-values are indicated as follows: * P < 0.05, ** P < 0.01, *** < 0.001, (no asterisk) = not significant.

### Implications for habitat fragmentation and mimicry

Human activity in Northeastern Brazil over the last five hundred years has generated substantial habitat fragmentation in this region [57]. Today, much of Brazil's Atlantic Forest is highly fragmented with few stretches of continuous forest [19]. Because *Heliconius *butterflies are relatively sedentary, we expect this extensive fragmentation to limit migration among habitat patches and potentially generate genetic differentiation among populations over time. Consistent with this expectation, our analyses revealed widespread genetic differentiation among populations of both *H. erato *and *H. melpomene*. While it seems likely that the observed genetic differentiation is at least partially related to extensive habitat fragmentation, future comparative work in undisturbed habitats will be required to quantify the exact impact of fragmentation relative to baseline differentiation among populations in a natural setting.

The extent of population subdivision across the range of *Heliconius *species has potentially important implications beyond estimating the impact of fragmentation on population connectivity. For instance, it has been proposed that novel color patterns may occasionally arise and become locally abundant as a result of genetic drift [49,51]. From there, these patterns may spread out to neighboring populations via a shifting balance type mechanism, thereby generating the geographic patchwork of wing pattern races which are particularly evident in *H. erato *and *H. melpomene *[51,58]. The baseline conditions necessary for this process to operate are small local population sizes and genetic drift, but allozyme data from a variety of *Heliconius *species have shown that populations are not genetically differentiated, arguing against local population genetic structure and drift. In contrast, a recent study revealed widespread genetic differentiation among *Heliconius *populations from Costa Rica based on AFLP data [43]. Here we have shown that population genetic differentiation is common in *Heliconius *and evident in microsatellites and mtDNA sequence data, in addition to AFLPs. If *Heliconius *populations are generally subdivided, independent of human induced habitat fragmentation, this may have allowed drift to contribute to the establishment of novel warning pattern phenotypes over their evolutionary history.

## Conclusions

Brazil's Atlantic Forest is a highly fragmented habitat and a priority for conservation efforts. Here we have shown that populations of two *Heliconius *butterfly species from the northern Atlantic Forest display widespread population genetic differentiation. These results are consistent with the expectation that fragmentation should reduce dispersal among neighboring habitat patches. The results also lend support to the observation that fine-scale population genetic structure may be common in *Heliconius*, which may have contributed to the evolution of mimetic diversity in the genus.

## Methods

### Sample collection

Between January 2007 and January 2008, we collected adult *Heliconius erato *and *Heliconius melpomene *specimens from various populations throughout the State of Rio Grande do Norte in northeastern Brazil (Figure 1). Our final sample set consisted of 136 *H. erato *from 8 locations and 146 *H. melpomene *from 7 locations (Table 5), with distances between sites ranging from 3 to 314 km. Seven sites were remnant patches of coastal Atlantic Forest and one was a cooler habitat island in the semi arid Caatinga scrub.

**Table 5:** Number of *H. erato *and *H. melpomene *specimens analyzed from the eight habitat fragments in Rio Grande do Norte, Brazil.

Site	GPS coordinates	*H. erato *(N)	*H. melpomene *(N)
MRT	6°5'5"S, 37°53'59"W	9	0
JND	5°53'23"S, 35°21'8"W	29	11
CTR	5°53'14"S, 35°13'9"W	20	21
CTL	5°54'25"S, 35°14'17"W	25	24
JQ	5°55'33''S, 35°11'42''W	23	23
FLN	6°4'59"S, 35°11'1"W	5	26
PP	6°13'23"S, 35°4'14"W	18	26
BF	6°24'49"S, 35°4'50"W	7	15

### Microsatellite analysis

Using eight individuals from each species, we performed an initial screen of fourteen microsatellite loci: nine (Hel02, Hel04, Hel05, Hel08, Hel10-13, Hel15) described by Flanagan *et al. *[59], four (Hm06, Hm08, Hm16, Hm22) described by Mavárez & González [60], and one (He-Ca-001) described by Tobler *et al. *[61]. The microsatellites that amplified consistently were selected for analysis (Table 6). PCR amplification was performed using standard conditions and reagents following Flanagan *et al. *[59] and Mavárez & González [60]. Fluorescent-labeled PCR products were separated with an ABI3730 Genetic Analyzer (Applied Biosystems, Foster City, CA.) and sized and scored using ABI GeneMapper software v. 3.7.

**Table 6:** Details of microsatellite loci genotyped in *H. erato *and *H. melpomene*.

				**T**_**a **_**(**^**o**^**C)**		
Locus	Primer sequence (5'-3')	Core repeat	GenBank number	*H. erato*	*H. melpomene*	**Ref**.
Hel02	F: TCAAAATGTTGCAGACCGAG	(GA)_13_(GAAA)_2_(GA)_2_	AF481467	55	-	[59]
	R: TGCACTTCATTGTAAGGCGT					
Hel05	F: TGCTGTCCATACCCAACTCA	(GA)_14_CA(GA)_4_	AF481470	52	55	[59]
	R: CGAACTCACAACCATCAGTCA					
Hel10	F: TCTCACTTTCCCACACAGCA	(CA)_7_TA(CA)_10_	AF481475	55	-	[59]
	R: TGTGAAGAGACACATGGGGA					
Hel11	F: TTTCTTTTGAGTCCCGATGG	(CA)_12_	AF481476	55	55	[59]
	R: ATCTCAGAACTGGTCGGCAG					
Hel12	F: CGGCACTTCATGTTTCATTT	(TAG)_4_	AF481477	55	-	[59]
	R: GGCATTTGACTTCAGAATGG					
Hel13	F: ATTTCATAGTAACGCCCTCC	(CA)_13_	AF481478	55	-	[59]
	R: TGACTTATCGCTAAGGTCAA					
Hm06	F: AAATAGTGTGCGGCGGAATA	(CA)_7_	DQ020077	55	-	[60]
	R: TGGAGTAGAAATGCGGGTTTA					
Hm08	F: AAAGCCTGAGTGCCGTATTG	(CA)_17_	DQ020079	-	55	[60]
	R: GCAATGTCAGCATCGAATGT					
Hm16	F: CGGATAGACATTTGTTAAAGTGTG	(CA)_14_	DQ020086	-	55	[60]
	R: ACGAGGATGCGGACTACG					
Hm22	F: CCTCGTCCAAACTCCAAAAC	(GA)_16_	DQ020090	-	52	[60]
	R: AACAATGTCACAACCATCGC					

For each locus we calculated standard population genetic statistics such as allele frequencies, expected heterozygosity (*H*_*E*_), observed heterozygosity (*H*_*O*_), and deviations from Hardy-Weinberg equilibrium using Arlequin 3.0 [62]. In addition, we used Arlequin 3.0 to estimate AMOVA-based [63] fixation indexes (F_ST_) and to perform Mantel tests, comparing population pairwise F_ST _values to straight line geographic distances, to test isolation-by-distance (IBD).

### AFLP analysis

We genotyped each individual with AFLPs using a plant mapping kit (Applied Biosystems) and separated fragments with an ABI3730 Genetic Analyzer. Three selective primer combinations were used to generate fragments: EcoRI-ACT/MseI-CAT, EcoRI-ACT/MseI-CTG and EcoRI-ACA/MseI-CTG. We separated fragments with an ABI3730 Genetic Analyzer and scored fragments between 50 and 500 bp using ABI GeneMapper software v. 3.7. For each species, we calculated AFLP gene diversity using the Bayesian method of Zhivotovsky [64]. We calculated F_ST _values and performed Mantel tests using Arlequin 3.0.

### Mitochondrial DNA analysis

We PCR amplified and sequenced a 1600 bp region of mitochondrial DNA from each individual using the primers and methods described by Béltran *et al. *[65]. This region spans the 3' end of COI, tRNA-Leu, and COII. Contigs were assembled with Sequencher 3.1 (Gene Codes Corporation, Ann Arbor, MI) and aligned by eye. Arlequin 3.0 was used to calculate nucleotide diversity (π) and F_ST _values as well as perform Mantel tests. DNA sequences were submitted to GenBank under accession numbers GU330064 - GU330070, GU330108 - GU330114, and HQ701917 - HQ702184.

## List of abbreviations

AFLP: amplified fragment length polymorphism; AMOVA: analysis of molecular variance; F_ST_: Wright's fixation index; msat: microsatellite; mtDNA: mitochondrial DNA; PCR: polymerase chain reaction; SNP: single nucleotide polymorphism.

## Authors' contributions

PAM, MZC and MRK planned the project; PAM and MZC collected specimens; PAM and SPQ generated molecular data; PAM and MRK analyzed the data; PAM, MZC and MRK wrote the paper; all authors read and approved the final manuscript.

## Acknowledgements

We thank Katia Scortecci (Genome Lab-UFRN) for support during the early stages of this project, Luis Vicente Burle Maciel for assistance with the map, and Durrell Kapan and reviewers for comments on the manuscript. Butterflies were collected under MMA (Brazil) permit #10894-1 to MZC. This project was funded by grants from CAPES and the Graduate Program in Ecology at UFRN to PAM, FAPERN/CNPq (PPP/2007) to MZC, and NIH NIGMS grant GM068763 to MRK.
